# P02.159. EEG asymmetry, coherence, and temperament in children

**DOI:** 10.1186/1472-6882-12-S1-P215

**Published:** 2012-06-12

**Authors:** Y Wang, Y Chen

**Affiliations:** 1 Institute of Natural Healing Sciences, NanHua University, DaLin, ChiaYi, Taiwan

## Purpose

Although neurofeedback has been used as one of the complementary and alternative medicine approaches, many EEG characteristics are still unknown. In order to develop a reliable protocol, it is necessary to establish a quantitative EEG normative database of the interested study group. Test of Nonverbal Intelligence (TONI) scores and 9 temperament dimensions, including activity level, predictability, approach/withdrawal, adaptability to change, intensity of reaction, threshold of responsiveness, general quality of mood, distractibility, and attention span/persistence were assessed.

## Methods

One hundred sixteen children of age 11 and 12 were recruited in this study. Each subject completed a Middle Childhood Temperament Questionnaire (MCTQ) and TONI and completed a 1-minute closed-eye EEG measurement of 14 electrodes. The subjects were separated medially and t-tests were conducted on 42 EEG asymmetry (7 interhemisphere pairs, each of 6 frequency ranges) and 91 EEG coherence variables of each frequency range (p<0.05).

## Results

The data affirmed that the majority of subjects were left rather than right active for alpha frequencies at frontal sites under closed-eye resting conditions. General quality of mood, determined by MCTQ, suggested higher frontal and parietal EEGs of right hemisphere are significantly related to more negative moods. No gender effect was observed among all measures of EEG asymmetry. EEG coherence, on the other hand, has identified significant differences between genders, along with activity level, general quality of mood, and TONI score. Boys generally had higher values in EEG coherence, a result consistent with an earlier study by Barry (2007).

## Conclusion

Our study suggested that not all children’s temperament dimensions are distinguishable by EEG asymmetry or EEG coherence. Among them, mood is the only factor that may be identified by inter-asymmetry. On the other hand, EEG coherence is likely a more sensitive parameter, and higher values in coherence may be related to low activity, negative mood and high TONI scores of these young students.

